# Unmasking Chaotic Attributes in Time Series of Living Cell Populations

**DOI:** 10.1371/journal.pone.0009346

**Published:** 2010-02-22

**Authors:** Michel Laurent, Jean Deschatrette, Claire M. Wolfrom

**Affiliations:** Laboratoire Dynamiques cellulaires et Modélisation, Unité Mixte de Recherche 8080, Centre National de la Recherche Scientifique, Unité Mixte de Recherche 757, Inserm, Université Paris-Sud, Orsay, France; University of Nottingham, United Kingdom

## Abstract

**Background:**

Long-range oscillations of the mammalian cell proliferation rate are commonly observed both *in vivo* and *in vitro*. Such complicated dynamics are generally the result of a combination of stochastic events and deterministic regulation. Assessing the role, if any, of chaotic regulation is difficult. However, unmasking chaotic dynamics is essential for analysis of cellular processes related to proliferation rate, including metabolic activity, telomere homeostasis, gene expression, and tumor growth.

**Methodology/Principal Findings:**

Using a simple, original, nonlinear method based on return maps, we previously found a geometrical deterministic structure coordinating such fluctuations in populations of various cell types. However, nonlinearity and determinism are only necessary conditions for chaos; they do not by themselves constitute a proof of chaotic dynamics. Therefore, we used the same analytical method to analyze the oscillations of four well-known, low-dimensional, chaotic oscillators, originally designed in diverse settings and all possibly well-adapted to model the fluctuations of cell populations: the Lorenz, Rössler, Verhulst and Duffing oscillators. All four systems also display this geometrical structure, coordinating the oscillations of one or two variables of the oscillator. No such structure could be observed in periodic or stochastic fluctuations.

**Conclusion/Significance:**

Theoretical models predict various cell population dynamics, from stable through periodically oscillating to a chaotic regime. Periodic and stochastic fluctuations were first described long ago in various mammalian cells, but by contrast, chaotic regulation had not previously been evidenced. The findings with our nonlinear geometrical approach are entirely consistent with the notion that fluctuations of cell populations can be chaotically controlled.

## Introduction

Fluctuations of cell proliferation have been observed in many types of normal or tumoral mammalian cells *in vitro* as well as *in vivo* [1]–[4], including blood and bone marrow cells [5], [6]. Such fluctuations are the result of a combination of deterministic regulation, through feedback loops, and stochastic influences, both internal (cell death) and external (environmental effects as well as noise due to data determination). The resulting fluctuations may be cyclic, as in so-called periodic diseases [7], [8], but the most frequent patterns are more or less irregular, both in frequency and amplitude. Assessing the predominantly stochastic or deterministic, possibly chaotic, nature of such short and irregular data sets is a difficult task and the subject of methodological debate. R.M. May's theoretical work in 1974 showed that simple deterministic rules may explain the complex fluctuations observed in population time series, with a broad spectrum of dynamics, from erratic, to periodic, to chaotic [9], [10]. The well-known Mackey-Glass model for the regulation of circulating white blood cell numbers also predicts various dynamics from stable, through periodically oscillating to a chaotic regime, depending on the duration of delays for the feedback signals [11], [12]. In fact, the various observed dynamics of biological systems, stochastic, periodic or chaotic, may be mixed or alternated in order to fulfill various biological purposes. Thus, discerning how long-range cell population fluctuations arise is a key issue for cell biologists, because these fluctuations play a critical role in physiology. For instance, they determine segmented embryo development [13], [1], episodic renewal of adult tissues, endocrine functions, tumor growth and metabolism. Detection of their possible chaotic nature may provide information about underlying feedback loops; it appeared to us, however, that there was no simple way of detecting chaos in small biological data sets. We previously designed a nonlinear analysis method, based on the recurrent representation of cell population data. Using this method, we detected a deterministic structure, an attracting fixed-point, in various time-series, both *in vitro* and *in vivo*. We detected such focal points in: i) the cultured rat liver cancer cell line Fao, which is clonal in origin [14]; ii) various series of primary cultures of mouse bone marrow cells, and in their derived blood progenitors [15]; and iii) circulating human neutrophils *in vivo*, in a patient presenting with a T-cell lymphoma [16]. In cultured cells, experimental culture conditions were kept as constant as possible. However two zeitgebers were clearly part of the dynamics, namely the medium change every other day, and the passaging of the cells every fifth, sixth, or seventh day (depending on the series) for hepatoma cells. For the blood cells in vivo, the spontaneous fluctuations were recorded at various time intervals, depending on the clinical context. In all these series, only horizontal sampling was considered. Our method of analysis showed that for some cell types the fluctuations of cell number were deterministic. However, nonlinearity and determinism are only necessary conditions for chaos, they do not by themselves constitute a proof of chaotic dynamics. Therefore, we used the same analytical method to analyze four well-known chaotic attractors: the Lorenz attractor, the Verhulst system, the Rössler system, and the Duffing oscillator. We found there was a structure similar to that observed in cell populations underlying the oscillations of certain coordinates of these four systems.

## Results

### Brief Explanation of the Method (Figure 1) and Experimental Background

**Figure 1:**
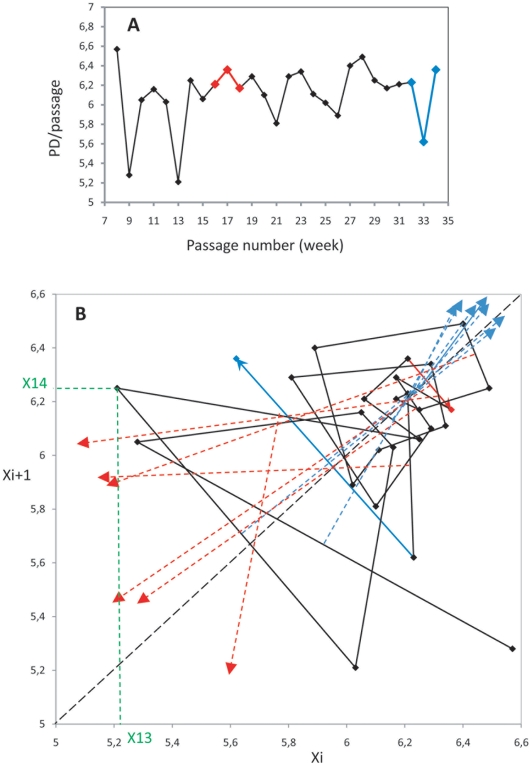
Method of map construction and analysis. **A**: segment of a curve of the proliferation rate of rat liver cancer cell line Fao: 27 consecutive 6-day passages in culture. X-axis: passage number; Y-axis: proliferation rate, expressed as population doublings/passage (PD/passage). **B**: the corresponding map is constructed by displaying the proliferation rate data in the recurrent form x_i+1_ versus x_i_ (*e.g.* the green lines x_i+1_ to x_i_ that construct the point corresponding to the segment p13-p14). The successive points on the map are joined together, as a succession of vectors. When x_i_ is a local peak (*i.e.* if x_i−1_<x_i_>x_i+1_), then the vector points south-east (highlighted in red). Whereas when x_i_ is a trough, (i.e. if x_i−1_>x_i_<x_i+1_), the vector points north-west in the plane (highlighted in blue). The bisecting line, i.e. the line perpendicular to the mid-point of the vector, is drawn for each trough (blue dotted arrows) and peak (red dotted arrows) vector. Coordination, if any, of the bisecting lines defines a fixed point, *i.e.* a point on the diagonal where x_i_ = x_i+1_ (which is therefore a stable level of cell growth), as shown here for convergence of the bisecting lines of trough vectors on coordinates 6.25/6.25. Note that there is no such coordination of the peak vectors in these cancer cells. (The complete analysis of the cell line was published in [14]).

The technical approach is explained here briefly (further details can be found in Methods and Appendix S1): 1) we represented the evolution of data by plotting x_i_ (the data at the i^th^ time-point) on the x-axis, *versus* x_i+1_ (data at the i+1^th^ time-point) on the y-axis. Let Mi be a point of coordinates (x_i_, x_i+1_). Consecutive points are joined. In this representation, if x_i_ is a local minimum, i.e. if x_i_<x_i−1_ and x_i_<x_i+1_, then the segment M_i_M_i+1_ runs from the south-east towards the north-west. Similarly, if x_i_ is a local maximum, *i.e.* if x_i_>x_i−1_ and x_i_>x_i+1_, then the segment M_i_M_i+1_ runs from the north-west towards the south-east; 2) we then drew the bisecting line (the line perpendicular to the vector, intersecting at its midpoint) for each vector on the map, to compare the orientation of the vectors illustrating the local minima (troughs) and the local maxima (peaks). Note that the geometric pattern is determined by only the numbers (x_i_, x_i+1_, x_i+2_…x_n_) and their order of succession, the time dimension being embedded in the map. The geometric pattern is thus independent of the regularity and size of the time intervals. This method was initially designed for analyses of long-term proliferation of various types of mammalian cells; these analyses revealed a deterministic pattern and identified how it depended on cell type. Briefly: in rat liver cancer cells, we observed that the bisecting lines of trough vectors converged on a high fixed point. However, in mouse blood progenitors the bisecting lines of peak vectors converged on a low fixed point. We found no regulation in proliferation data from dedifferentiated or embryonic cells, and we observed a dual control in proliferation data from normal mouse bone marrow cells, and from normal human fibroblasts (however, the latter was a short series). Calculation of means and variances for all scattered points of intersection also confirmed that this convergence, *i.e*. the distribution of a_i_ when present, was deterministic in nature. Monte-Carlo simulations were used to validate these initial findings [14], [15].

### Behavior of Known Chaotic Systems

**Figure 2:**
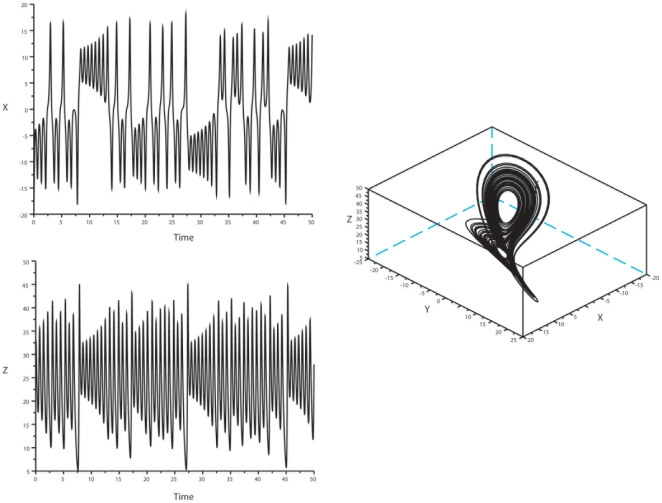
Lorenz oscillator: oscillatory patterns and phase-space representation. Lorenz attractor standard values for the constants were set as follows:
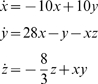

**Figure 3:**
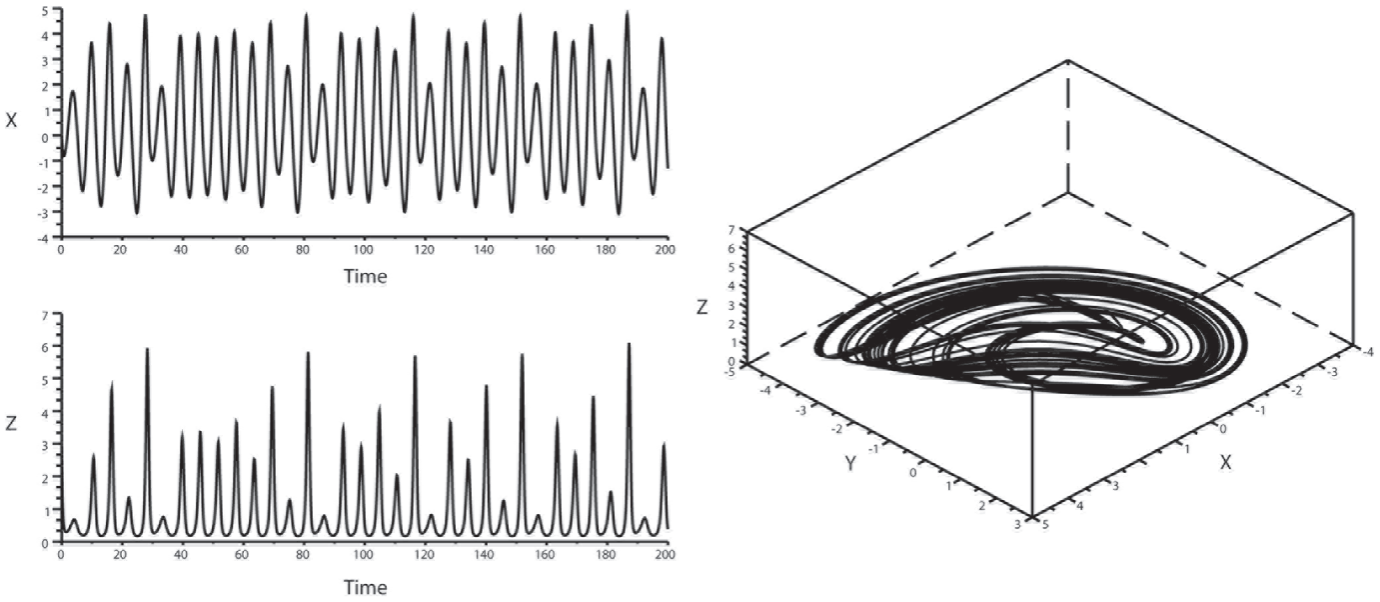
Rössler system: oscillatory patterns and phase-space representation. Rössler system standard values for the constants were set as follows:
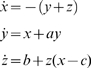
with a = 0.398, b = 1, c = 3. *Left panel*: Ordinate: oscillatory behavior of variables x (top) and z (bottom). Abscissa: time. *Right panel*: phase-space representation of the attractor.

**Figure 4:**
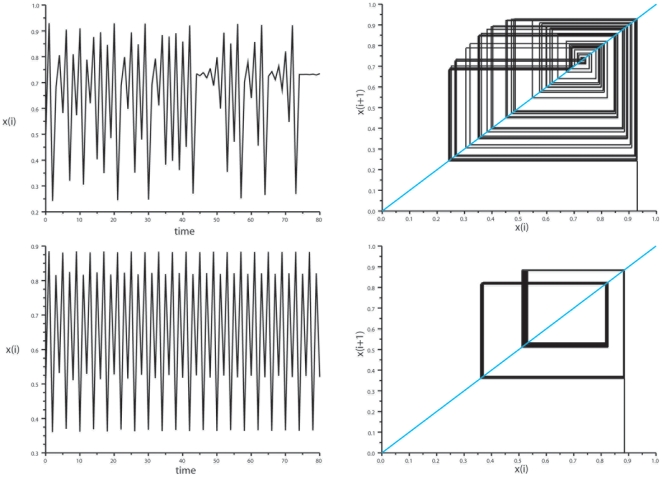
Verhulst system: oscillatory pattern and phase-space representation. Verhulst system standard values for the constants were set as follows:
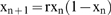
with r = 3.72. *Left panel*: Ordinate: oscillatory behavior of variable x. Abscissa: time. *Right panel*: phase-space representation of the attractor. Chaotic (top) and birhythmic (bottom) conditions are compared.

**Figure 5:**
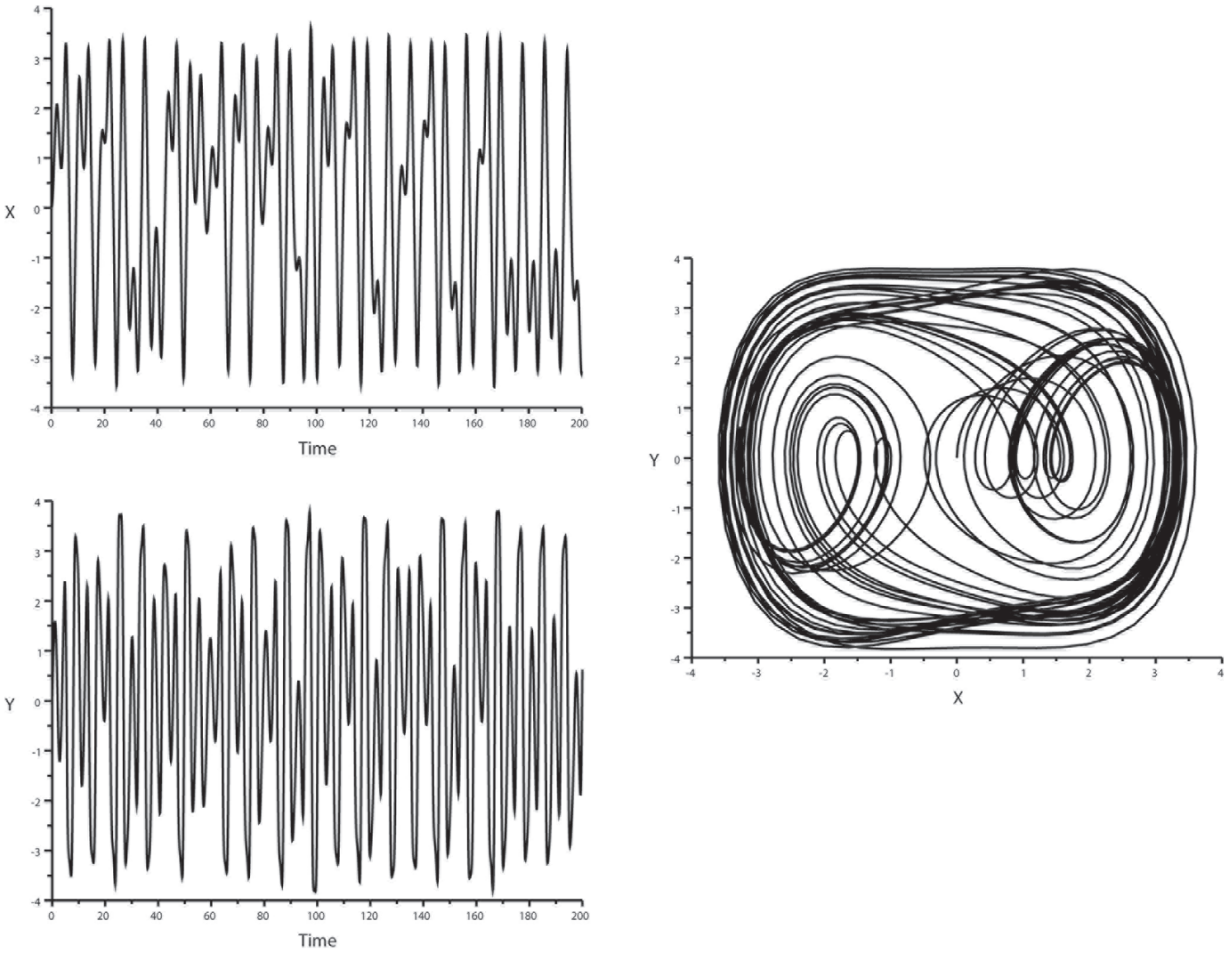
Duffing oscillator: oscillatory patterns and phase-space representation. Duffing oscillator standard values for the constants were set as follows:
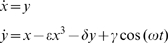
with 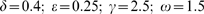. *Left panel*: Ordinate: oscillatory behavior of variables x (top) and y (bottom). Abscissa: time. *Right panel*: phase-space representation of the attractor.

We examined the changes over time of four well-known low-dimensional chaotic systems: Lorenz, Rössler, Verhulst, and Duffing. The Lorenz system was designed for convection analysis and is not generally used to study population data [17] (Figure 2). The Rössler model was initially proposed in different settings, but has also been used to model thermodynamics and changes in the population [18], [19] (Figure 3). The Verhulst model was specifically designed to analyze and model biological populations [20] (Figure 4). The Duffing oscillator has been found appropriate for a wide variety of biological functions, when they include damping and regular forcing of the dynamics (Figure 5). The constants of the equations for each system were set at values resulting in typical chaotic behavior, and the map was drawn for variables x, y and z.

*Left panel*: Ordinate: oscillatory behavior of variables x (top) and z (bottom). Abscissa: time. *Right panel*: phase-space representation of the attractor.

**Figure 6:**
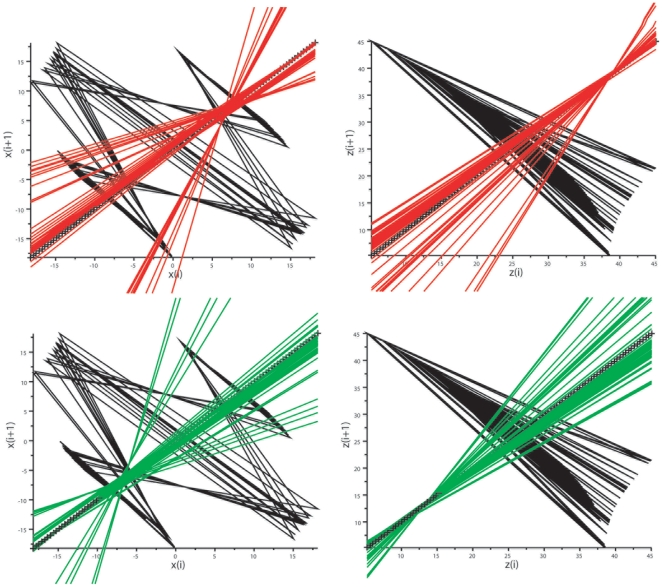
Lorenz oscillator: analysis of the map x_i_ *vs* x_i+1_. *Left panel:* the bisecting lines of the trough vectors (top, red lines) and of the peak vectors (bottom, green lines) for variable x. *Right panel:* the bisecting lines of the trough vectors (top, red lines) and of the peak vectors (bottom, green lines) for variable z. Note the focal concentration of maxima bisecting lines (at coordinates 12.5/12.5) and minima bisecting lines (at coordinates 40/40) for variable z The focal concentration of bisecting lines for the variable x is slightly less precise.

**Figure 7:**
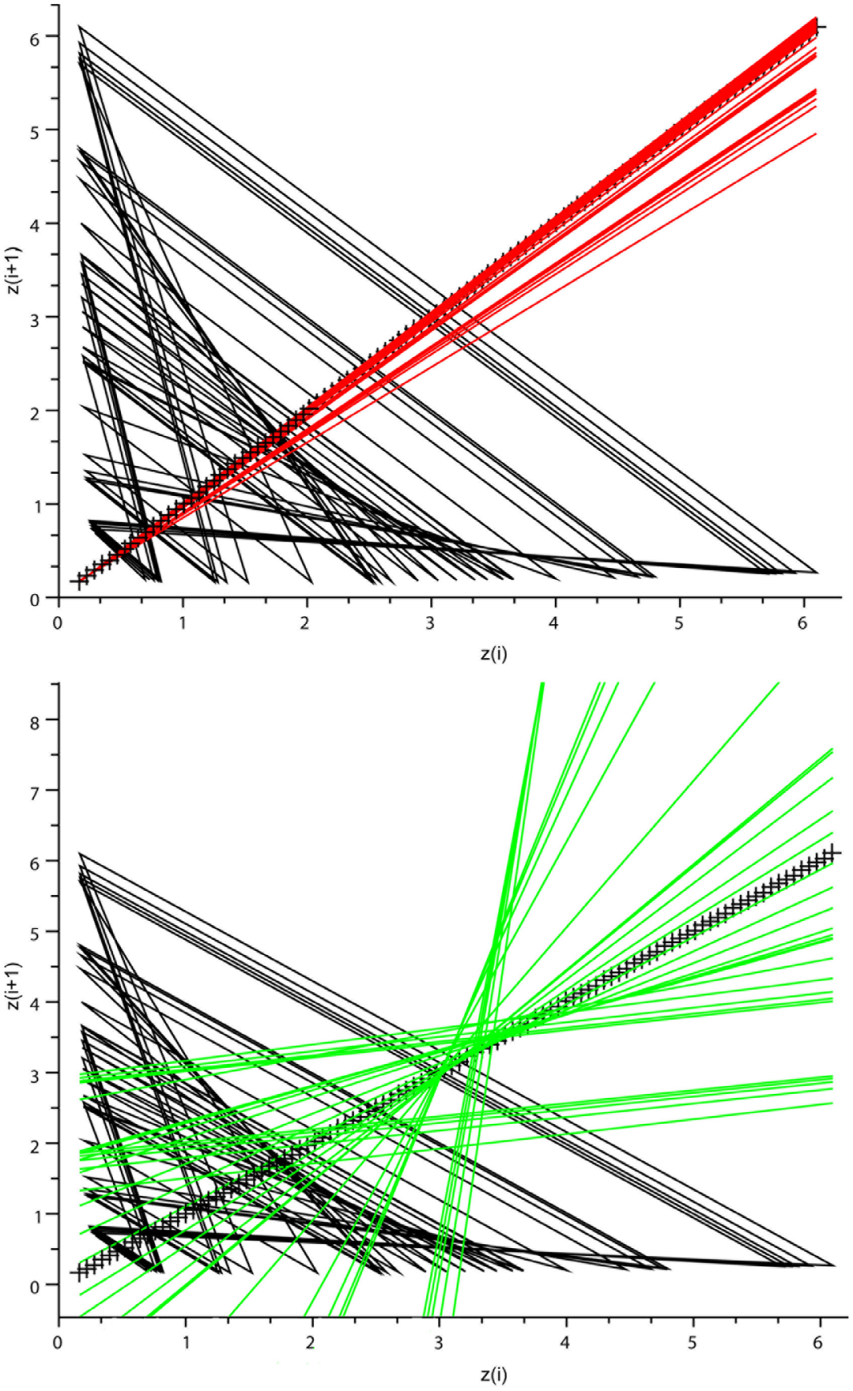
Rössler system: analysis of the map x_i_ *vs* x_i+1_. *Top*: bisecting lines (red) for the trough vectors. *Bottom*: bisecting lines for the peak vectors (green lines). There is a characteristic convergence of the bisecting lines of local maxima on the map. In contrast, the local minima are not coordinated.

**Figure 8:**
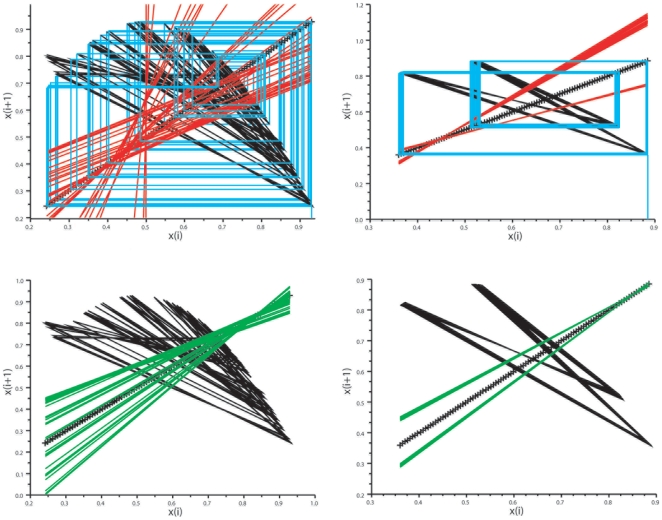
Verhulst system: analysis of the map x_i_ *vs* x_i+1_. *Left panel* displays the maps for chaotic conditions. *Top*: bisecting lines of the peak vectors (red lines). *Bottom*: bisecting lines of the trough vectors (green lines). Note the convergence of bisecting lines of local minima. The local maxima were not coordinated with this set of parameters. *Right panel*: maps for birhythmic dynamics. *Top*: note the two narrow bundles of superimposed bisecting lines of the two types of peak vectors (red lines). *Bottom*: note the two narrow bundles of the superimposed bisecting lines of the two types of peak vectors (green lines).

**Figure 9:**
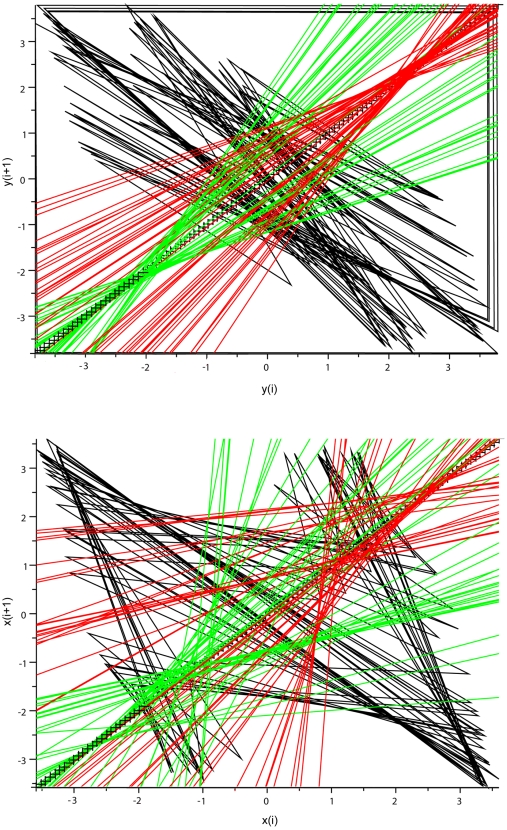
Duffing oscillator: analysis of the map x_i_ *vs* x_i+1_. *Top*: map for the variable y. *Bottom*: map for the variable x. Bisecting lines for the vectors of peaks are in green, bisecting lines for the vectors of troughs are in red. Note dual control of local maxima and minima for the variable y, with convergence of bisecting lines of peaks on a low fixed-point (coordinates about −2/−2) and convergence of local minima on a high-fixed point (coordinates about 2/2). In contrast the bisecting lines of the peak and trough vectors of the variable x are disordered.

The results were as follows: 1) *Lorenz attractor*: Considering variable y, no coordination was noted for the bisecting lines of local maxima and minima in the Lorenz system with this set of parameters. However, focal concentration of maxima and minima bisecting lines was clearly observed for variable z (Figure 6), and also for variable x, albeit in a slightly more dispersed manner. 2) *Rössler system*: we observed a characteristic convergence of the bisecting lines of local maxima on the map for variable z only, whereas the local minima of z were not coordinated. The local maxima and minima of x and y were not coordinated. This determinism of peaks for z was confirmed with two other sets of values for the constants (b = 1.5 and 2, with a and c unchanged) (Figure 7). 3) *Verhulst system*: we observed a convergence of bisecting lines of local minima. The local maxima were not coordinated with this set of parameters (Figure 8). 4) *Duffing oscillator*: It displayed a dual control of local maxima and minima, with convergence of bisecting lines of peaks on a low fixed-point and convergence of local minima on a high-fixed point, for variable y, with the damping coefficient δ = 0.4; however, this coordination disappeared when there was no damping of the system (δ = 0). There was no focalization of the bisecting lines for variable x (Figure 9).

### Comparison with Other Dynamics

**Figure 10:**
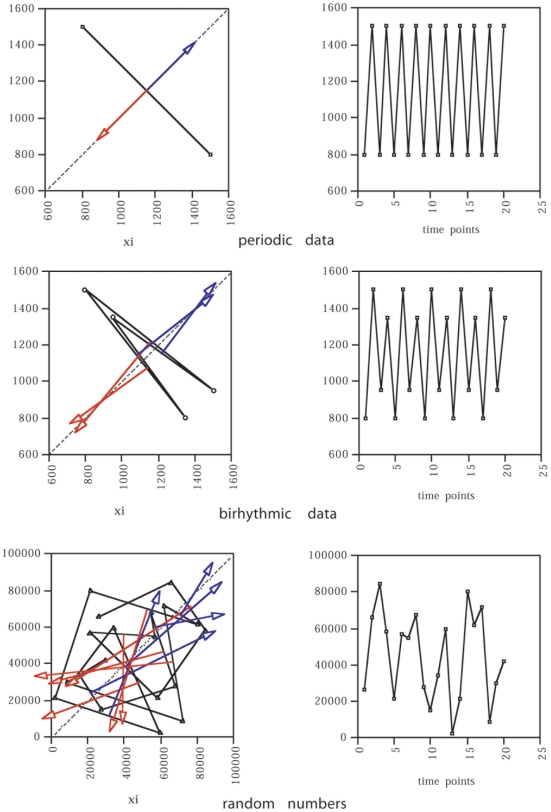
Comparison with other dynamics. *Left panel*: return maps, *Right panel*: oscillatory behaviors. *Top*: sinusoidal oscillations. The vectors for local minima and local maxima are superimposed on one line perpendicular to the diagonal, and their bisecting lines are superimposed on the diagonal, and oriented upward for the local minima, and downward for the local maxima. *Middle*: birhythmic oscillations. There are two vectors representing all local maxima, the bisecting lines of which intersect the diagonal at a low fixed point, and two vectors representing all local minima, the bisecting lines of which intersect the diagonal at a high fixed point. *Bottom*: random numbers; the bisecting lines of the vectors are dispersed.

Examples of sinusoidal, birhythmic, and stochastic dynamics using the same analytical approach are illustrated in Figure 10. i) In the case of sinusoidal oscillations (Figure 10 top), the vectors for local minima and local maxima are superimposed on one line perpendicular to the diagonal, and their bisecting lines are superimposed on the diagonal, and oriented upward for the local minima, and downward for the local maxima. ii) In the case of birhythmic oscillations (Figure 10 middle), there are two vectors representing all local maxima, the bisecting lines of which intersect the diagonal at a low fixed point, and two vectors representing all local minima, the bisecting lines of which intersect the diagonal at a high fixed point. When a small amount of noise, such as the variability due to sampling imprecision hampering the perfect localization of a local maximum or minimum, is included in a birhythmic system, the bisecting lines appear as two narrow bundles of lines rather than two single lines, e.g. the birhythmic Verhulst system in Figure 8, right. iii) When stochasticity predominates (Figure 10 bottom), the bisecting lines of the vectors are dispersed. Monte-Carlo analysis of previous experimental series strengthened these findings (see Appendix S1), and confirmed that the method could discriminate between chaotic and nonchaotic dynamics.

## Discussion

### Analysis of the Topological Results

We found that convergence on a fixed point of the lines bisecting maxima and minima vectors was a common characteristic of low-dimensional chaotic oscillators. This is consistent with our hypothesis that this converging structure in the phase-space representation of certain cell proliferation time series indicates a chaotic behavior. A seminal work in 1980 by Packard and co-workers demonstrated that reconstruction using a single coordinate of a dynamic system provides an accurate image of the attractor. Using the Rössler system, Packard and co-workers showed that the state of the system at any given time could be specified by measuring any three independent quantities, including, for instance, the value of a coordinate at three successive times [21]. Our construction of the map defining vectors for local maxima and minima of cell population data follows this principle, using the three consecutive data defining each local maximum and each local minimum. As a second step, we added a projective transformation using the bisecting lines of the vectors. In the computer maps we generated, bisecting line focalization was observed for at least one coordinate of each system. The reasons for different coordinates being involved and for a selective convergence on high (Verhulst) or low (Rössler) or both (Duffing) fixed points are unclear at present and require further analysis. Preliminary tests varying the equation parameters showed that the pattern of convergence remained unaffected, except for the Duffing oscillator which lost the converging pattern when the damping coefficient was deleted from the equation.

### The Problem of Discriminating Chaos and Noise

A critical issue is to be able to affirm that the observed convergence of bisecting lines in various proliferation series is a consequence solely of chaotic dynamics; this is particularly important because there always are some stochastic components in experimental data. In our previous experimental observations, we checked the dispersion (means and sd) of the intersections, and used the Monte-Carlo technique of surrogate data to help distinguish between random and deterministic fluctuations [14]–[16, and Appendix S1]. Mathematicians in the field exploit various techniques of analysis involving either surrogate data [22], [23], or the forecasting error technique [24], or the confinement technique [25], with extended series of data to affirm the chaotic or stochastic nature of fluctuations. At this stage of our work, we are unable to adapt these advanced techniques to our small experimental samples. Instead, we focused on the fact that the bisecting lines of the vectors of peaks and troughs displayed a similar structure in known chaotic systems and in various experimental cell population series; we interpreted this common behavior as indirect evidence of common chaotic dynamics. We believe that this novel approach has two advantages: it is a new simple method of analysis applicable to small series and can therefore be exploited by cell biologists; and it is a graphical empirical approach independent of the stringent mathematical criteria of other methods. However, we hope that the continuing dialogue between cell biologists and mathematicians in the field of chaos 1) will allow comparison with other methods of analysis, and 2) will clarify the mathematical basis of the convergence of peak and trough bisecting lines in chaotic models and in experimental series.

### Relationship with Mammalian Cell Dynamics

Identifying the attractor for the dynamics of experimental data concerning mammalian cell proliferation requires further study. It may not be one of these four classical systems and may not even be the same for all mammalian cell types. With the current state of knowledge, the commitment of peaks and/or troughs of mammalian cell proliferation to two fixed points, low and/or high respectively, is evocative of a double-well control [26], [27] around two stable levels of growth, one minimum, one maximum. This bipolar control of cell proliferation, the net result of coordinated physiological regulators, presumably varies according to cell type. There are numerous positive regulators of cell growth, including growth factors, metabolic resources, oncogenes, and the telomere repair system. There are also various negative factors, which include contact inhibition, exhaustion of resources and cell senescence. In cultured cells, additional driving forces, which are also zeitgebers, are provided by periodic feeding and passaging of the cells. Depending on so-called initial conditions, including the net result of all regulatory factors and the cell type, the dynamics of cell proliferation may highlight the predominance of a ground-state/dampened level or a high/stimulated level, or both. We previously observed a high fixed point coordinating the local minima of growth of liver cancer cells, which are deregulated to grow very rapidly. However, a low fixed point organized the growth maxima of bone marrow progenitors, which must be preserved as a reservoir of stem cells.

Various characteristics of chaotic dynamics -near-periodic recurrence, adaptability, robustness, bounded amplitude and synchronization- are required for the persistent growth of tumor cells or to maintain blood progenitors. In contrast, dedifferentiated liver cells and undifferentiated embryonic cells displayed no coordination at all, consistent with their undetermined fate [14], [15]. The Mackey-Glass model fits all these cell behaviors well, as it predicts various dynamics, from stable through periodically oscillating to a chaotic regime, depending on the delays for the feedback signal [6], [11]. Observations by the Mackey group [8] on chronic cyclical neutropenias and leukemia illustrate the periodic regime. The occurrence of small stochastic fluctuations, i.e. stability with noise or simple randomness, was described long ago for various cells [3], [5]. We believe that the feedback loops in various cells can result in chaotic fluctuations of proliferation.

### Conclusions

For cell biologists, analysis of the regulatory loops which regulate cell proliferation is always fragmentary. There are three major advantages of the topological analysis we describe: i) the phase-space portrait provides an image of the long-term evolution of the cell population, ii) the bisecting lines of maxima and minima vectors help differentiate chaotically controlled or stochastic dynamics, and iii) the identification of the fixed level of growth governing the system. This graphical approach is very easy to use with experimental data from cell proliferation, and remains accurate even when intervals of data determination vary moderately, as the time dimension is embedded in the representation of the data.

We show here that the oscillations of four classical low-dimensional chaotic oscillators: Lorenz, Rössler, Verhulst and Duffing displayed a similar focal structure for the maxima and minima of oscillations on their phase space representation. This structure was also similar to the focal structure previously observed for various cell proliferation time-series. In the absence of direct classical proof of chaotic characteristics in small experimental series, we believe that this similar behavior confirms the validity of the approach to detect chaotic behavior in cell proliferation data. However, further mathematical studies are required to determine 1) why this convergence of the bisecting lines occurs in chaotic settings, and 2) the statistical limitations of the sampling in this analytical approach. From a practical point of view, knowledge of underlying chaotic dynamics is critical to the analysis of various mammalian cell functions related to proliferation rate, including metabolic pathway activity, telomere homeostasis [28], [29], and gene expression [13]. It is also of use in appraisal of tumor growth rate and prediction of anticancer drug efficiency [30], [31]. It may provide a basis for developing new ways of controlling the long-term dynamics of cell populations.

## Methods

### Construction of the Map and Bisecting Line Analysis (Figure 1)

**Step 1**: The map was constructed by displaying the cell proliferation data [in this figure we used the derivative cell proliferation rate, expressed as population doublings/passage (week)] in the recurrent form x_i+1_ versus x_i_. The complete analysis of the dynamics of this rat liver cancer cell line Fao can be found in [14]. In this representation, each segment of the corresponding proliferation curve (Figure 1 top), from one value to the next, is thus defined as a point on the map, with the value on the x-axis being x_i_, and on the y-axis, x_i+1_. **Step 2**: The successive points were joined together (Figure 1 bottom), showing the long-term changes over time of the cell population as a succession of orbits. The successive phases of the proliferation curve: ascending, local maximum (peak), descending, local minimum (trough), are further defined on this map as vectors. If x_i_ is a local peak [*i.e.* x_i−1_<x_i_>x_i+1_], then the vector points south-east. Likewise, if x_i_ is a trough, [*i.e.* x_i−1_> x_i_ <x_i+1_], then the vector points north-west. **Step 3**: the bisecting line (i.e. the perpendicular line crossing the mid-point of the vector) for each vector was drawn, to compare the orientation of the vectors illustrating all local minima, or local maxima, or ascending and descending segments. This paper analyzes the characteristic patterns that were obtained for the local maxima and minima. **Step 4**: the deterministic structure, if present, appears as the convergence of all bisecting lines of either the local minima, or the local maxima, on a fixed point, *i.e.* a point on the diagonal of the map where coordinates x_i_ and x_i+1_ are equal (this determinism for proliferation data of the rat liver cancer cell line (Fao), over twenty-seven weekly passages in culture, is shown in Figure 1). The fixed-point may be considered as a center of rotation of the attractor. The coordinates (a, a) of the fixed point, by construction, correspond to the mean of the x_i−1_ and x_i+1_ values which define all local minima (for high fixed point) or local maxima (for low fixed point).

Proof: let x_i_ be a local minimum. As the distances from the first point and the last point defining each vector to the fixed point on the diagonal are equal, we have:
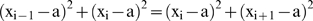
from which we derive the mathematical law of the fixed point value:
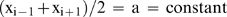



### Characteristics of the Method

The sampling was done at local extrema only for the continuous systems, in these theoretical computer-made time-series. In these series, which fluctuate from peak to trough without intermediate values, the level of the fixed point, if present, is equal to the mean of local maxima (for low fixed point) or local minima (for high fixed point). For the experimental data from living cell proliferation time-series, the sampling frequency corresponded to the full development of a batch of cells (*e.g.* about six days for cultured cancer cells), and therefore our estimate was that the frequency of the measured fluctuations was close to the frequency of the underlying fluctuations. In fact, we observed much more variation in the amplitude of the fluctuations of cell proliferation than in their frequency. However, there were some intermediate data between peaks and troughs, which appeared on the map as vectors pointing either north-east (ascending segments of the proliferation curve) or south-west (descending segments of the proliferation curve). These intermediate segments were analyzed separately. Therefore, in the experimental series, the value of the fixed point may be more or less close to the means of local extrema, depending on the number of intermediate data points between the local maximum and minimum values.

Cell biologists generally have to analyze small and sometimes irregular data sets for studies of the proliferation of living cells. Our previous experimental studies included 25–50 data series (corresponding to up to one year of weekly determinations) with data collected under strictly controlled experimental conditions for *in vitro* series; for *in vivo* series data is uneven and collected in uncontrolled conditions, with sampling being horizontal. Using preliminary empirical conditions, we selected series including at least eight peaks and eight troughs, without missing points, and considered that there was convergence if eighty to one hundred percent bisecting lines converged on the diagonal of the map for cell number peaks or troughs or both [12]–[14]. Clearly, further mathematical analysis is required to determine whether these preliminary conditions are optimal for such analyses.

## Supporting Information

Appendix S1 (0.03 MB DOC)
