# Supplementary material for: Unmasking Chaotic Attributes in Time Series of Living Cell Populations
Source: PLoS One. 2010 Feb 22;5(2):e9346. doi: 10.1371/journal.pone.0009346 (PMC2825257; doi:10.1371/journal.pone.0009346)
Supplement: Appendix S1 — (0.03 MB DOC) [file pone.0009346.s001.doc]

**Appendix-S1**

In our previously published experimental series, we used two statistical tests for the control mechanisms for fixed points of the dynamics of hepatoma cells and bone marrow progenitors [14,15]:

1) We wrote a computer program to determine the intersections of the bisecting lines of the trough vectors, by successively pairing all these vectors in each series. The mean and variance of the x and y co-ordinates of all scattered points of intersection were calculated. The same was done with the vectors for local maxima. This calculation confirmed the strong convergence of the vectors of local minima of hepatoma cells on a point located on the diagonal of the map, i.e. a fixed-point. The same confirmation was obtained for the two fixed points of marrow progenitor dynamics.

2) We used a Monte Carlo procedure to show that relationship (A) results from a deterministic control. Let y_i_ = (x_i-1_+x_i+1_)/2. We calculate the quantities y_1_, y_2_, ..., y_p_ corresponding to the different minima of the series and test the hypothesis that the set y_i_ is a constant, i.e., is of variance (or standard deviation, sd) zero. Let s_0_ be the std of the set (y_i_). We performed a random perturbation of the order of the series x_i_ then identified the local minima and calculated the quantities y_k_ of the perturbed (uncontrolled) series. Let s_1_ be the sd of these new y_k_. By repeating this procedure, we calculated the new sd’s s_2_, s_3_, ..., of several new series, obtained each time from the initial series by randomly shuffling the order. Our hypothesis is highly probable if only a very small number of the s_i_ with i=1, 2, …, are smaller than the observed valued s_0_. The same calculations were performed for the local maxima. The same method was applied for all series of data. However, we explicitly reported calculations only 1) for the 29-week series of bone marrow cultures: the probability of s_i_ ≤ s _0_ was 0.008; and 2) for the 6-9 months culture series of hepatoma cells: only 1.5% of s_i_ were ≤ than s_0_. So, the probability that a random sequence gives a s_i_ more extreme than the observed s_0_ is very small, and we may have confidence that the law (A) expresses a deterministic structure. The other time-series were shorter, or lacked one or two time points, and we chose to use the longest series in these two studies [14, 15].
